# Criteria for the use of omics-based predictors in clinical trials: explanation and elaboration

**DOI:** 10.1186/1741-7015-11-220

**Published:** 2013-10-17

**Authors:** Lisa M McShane, Margaret M Cavenagh, Tracy G Lively, David A Eberhard, William L Bigbee, P Mickey Williams, Jill P Mesirov, Mei-Yin C Polley, Kelly Y Kim, James V Tricoli, Jeremy MG Taylor, Deborah J Shuman, Richard M Simon, James H Doroshow, Barbara A Conley

**Affiliations:** 1 Biometric Research Branch, Division of Cancer Treatment and Diagnosis, National Cancer Institute, National Institutes of Health, Room 5W130, MSC 9735, 9609 Medical Center Drive, Bethesda, MD 20892-9735, USA; 2 Cancer Diagnosis Program, Division of Cancer Treatment and Diagnosis, National Cancer Institute, National Institutes of Health, Room 4W432, MSC 9730, 9609 Medical Center Drive, Bethesda, MD 20892, USA; 3 Cancer Diagnosis Program, Division of Cancer Treatment and Diagnosis, National Cancer Institute, National Institutes of Health, Room 4W420, MSC 9730, 9609 Medical Center Drive, Bethesda, MD 20892, USA; 4 Department of Pathology and Lineberger Comprehensive Cancer Center, Brinkhous-Bullitt Bldg., Campus Box 7525, University of North Carolina, Chapel Hill, NC 27599, USA; 5 Department of Pathology and University of Pittsburgh Cancer Institute, Hillman Cancer Center, UPCI Research Pavilion, Suite 2.32b, 5117 Centre Avenue, Pittsburgh, PA 15213, USA; 6 Frederick National Laboratory for Cancer Research, National Cancer Institute, National Institutes of Health, Bldg. 320, Room 2, 1050 Boyles Street, Frederick, MD 21702, USA; 7 Computational Biology and Bioinformatics, Broad Institute of Massachusetts Institute of Technology and Harvard University, 7 Cambridge Center, Cambridge, MA 02142, USA; 8 Biometric Research Branch, Division of Cancer Treatment and Diagnosis, National Cancer Institute, National Institutes of Health, Room 5W638, 9609 Medical Center Drive, Bethesda, MD 20892, USA; 9 Cancer Diagnosis Program, Division of Cancer Treatment and Diagnosis, National Cancer Institute, National Institutes of Health, Room 4W430, 9609 Medical Center Drive, Bethesda, MD 20892, USA; 10 Cancer Diagnosis Program, Division of Cancer Treatment and Diagnosis, National Cancer Institute, National Institutes of Health, Room 3W526, 9609 Medical Center Drive, Bethesda, MD 20892, USA; 11 Department of Biostatistics, University of Michigan, 1415 Washington Heights, Ann Arbor, MI 48109, USA; 12 Office of the Director, Division of Cancer Treatment and Diagnosis, National Cancer Institute, National Institutes of Health, Room 3A44, 31 Center Drive, Bethesda, MD 20892, USA; 13 Biometric Research Branch, Division of Cancer Treatment and Diagnosis, National Cancer Institute, National Institutes of Health, Room 5W110, 9609 Medical Center Drive, Bethesda, MD 20892, USA; 14 Cancer Diagnosis Program, Division of Cancer Treatment and Diagnosis, National Cancer Institute, National Institutes of Health, Room 4W426, 9609 Medical Center Drive, Bethesda, MD 20892, USA

**Keywords:** Analytical validation, Biomarker, Diagnostic test, Genomic classifier, Model validation, Molecular profile, Omics, Personalized medicine, Precision Medicine, Treatment selection

## Abstract

High-throughput ?omics? technologies that generate molecular profiles for biospecimens have been extensively used in preclinical studies to reveal molecular subtypes and elucidate the biological mechanisms of disease, and in retrospective studies on clinical specimens to develop mathematical models to predict clinical endpoints. Nevertheless, the translation of these technologies into clinical tests that are useful for guiding management decisions for patients has been relatively slow. It can be difficult to determine when the body of evidence for an omics-based test is sufficiently comprehensive and reliable to support claims that it is ready for clinical use, or even that it is ready for definitive evaluation in a clinical trial in which it may be used to direct patient therapy. Reasons for this difficulty include the exploratory and retrospective nature of many of these studies, the complexity of these assays and their application to clinical specimens, and the many potential pitfalls inherent in the development of mathematical predictor models from the very high-dimensional data generated by these omics technologies. Here we present a checklist of criteria to consider when evaluating the body of evidence supporting the clinical use of a predictor to guide patient therapy. Included are issues pertaining to specimen and assay requirements, the soundness of the process for developing predictor models, expectations regarding clinical study design and conduct, and attention to regulatory, ethical, and legal issues. The proposed checklist should serve as a useful guide to investigators preparing proposals for studies involving the use of omics-based tests. The US National Cancer Institute plans to refer to these guidelines for review of proposals for studies involving omics tests, and it is hoped that other sponsors will adopt the checklist as well.

## The promise of omics profiling for therapeutic decision making

High-throughput ?omics? technologies may allow more informative characterization of disease to better predict both an individual patient?s clinical course and the degree of benefit he or she may derive from new and existing therapies. The potential for detailed characterization of disease has been met with particularly great enthusiasm in oncology, where the heterogeneous character of malignant diseases has long presented challenges. However, despite the widespread use of these technologies in both preclinical and retrospective studies, it has proven more difficult than expected to translate their promise into clinically useful tests that can be used to guide management decisions.

This paper focuses on molecular tests derived from high-throughput omics assays (?omics-based tests? or simply ?omics tests?) as defined in the Institute of Medicine (IOM) report *Evolution of Translational Omics*[1]. An IOM committee that was convened to review omics-based tests for predicting patient outcomes in clinical trials defines ?omics? as the study of related sets of biological molecules in a comprehensive fashion. Examples of omics disciplines include genomics, transcriptomics, proteomics, metabolomics, and epigenomics. Further, the IOM committee defined an omics-based test as ?an assay composed of or derived from multiple molecular measurements and interpreted by a fully specified computational model to produce a clinically actionable result? [1].

A distinguishing characteristic of the omics tests discussed here is that computational methods are applied to the high-dimensional data to build mathematical models, often from a subset of the measured variables that have been identified through data-driven selection. This is in contrast to molecular tests based on pre-specified, biologically driven variables, such as mutations in genes targeted by a new therapeutic agent, which might be used to screen patients for eligibility for a clinical trial. Although these biologically driven tests must be based on assays with appropriate analytical performance, they are not subject to all of the same pitfalls that are inherent in omics tests involving complex computational models, so they are not the main focus of this paper.

The development path from high-throughput omics technology to a clinical-grade omics test requires rigorous attention to criteria including the following:

??Availability and quality of appropriate clinical specimens

??Requirements for the analytical performance of the omics assay

??Methods for omics data pre-processing

??Development of the mathematical predictor model and assessment of its performance

??Clinical interpretation of the test result

??Design of the clinical trial

??Ethical, legal, and regulatory issues

Given the rich data emerging from cancer genomics research, it might seem surprising that relatively few omics tests have successfully navigated this path to clinical use. In some cases, the use of omics tests in clinical trials, or their promotion for routine clinical use, has been premature [1].

There are many reasons for the paucity of omics tests that are able to provide patients and clinicians with information that is useful in the assessment and treatment of disease. They include difficulty in obtaining a sufficient number of acceptable-quality biospecimens with the desired clinical and pathological characteristics, as well as technical challenges in the development and implementation of assays that can be successfully applied to the available types of clinical specimens. Optimal analytical performance and reproducibility of an omics assay may be difficult to achieve, or the assay may lack robustness to ancillary pre-analytical influences on specimens. Translation has also been hampered by the difficulties in properly evaluating the accumulated body of evidence for an omics test by the time there is interest in its definitive evaluation in a clinical trial. Omics studies are often not reported in sufficient detail to allow assessment of the rigor with which the test was developed or evaluated. Adequate data and computer code are not always made available to allow understanding of the methods used in the development of the test or to facilitate independent replication of the results. Subtle flaws in statistical approaches for developing or assessing the performance of mathematical models may go undetected. It is essential to consider all of these issues before launching into a clinical study using an omics test in a way that might influence the clinical management of patients [2].

Presented here are criteria that should be addressed to determine the readiness of an omics test for use in a prospective clinical trial or study. These criteria apply to tests derived from high-dimensional data generated by any type of omics technology when those tests are being proposed for use in a study in a way that will influence patient care. The criteria address not only the strength of evidence in support of an omics test but also the practical issues that must be considered before using the test in a clinical setting. Commentary accompanies each criterion to provide the rationale for and more specific details about the type of information requested. The complete set of criteria is assembled into a checklist, which is shown in Table?1.

**Table 1:** Criteria for the use of omics-based predictors in National Cancer Institute-supported clinical trials

**Domain**	**Criteria**
Specimen issues	1. Establish methods for specimen collection and processing and appropriate storage conditions to ensure the suitability of specimens for use with the omics test.
	2. Establish criteria for screening out inadequate or poor-quality specimens or analytes isolated from those specimens before performing assays.
	3. Specify the minimum amount of specimen required.
	4. Determine the feasibility of obtaining specimens that will yield the quantity and quality of isolated cells or analytes needed for successful assay performance in clinical settings.
Assay issues	5. Review all available information about the standard operating procedures (SOPs) used by the laboratories that performed the omics assays in the developmental studies, including information on technical protocol, reagents, analytical platform, assay scoring, and reporting method, to evaluate the comparability of the current assay to earlier versions and to establish the point at which all aspects of the omics test were definitively locked down for final validation.
	6. Establish a detailed SOP to conduct the assay, including technical protocol, instrumentation, reagents, scoring and reporting methods, calibrators and analytical standards, and controls.
	7. Establish acceptability criteria for the quality of assay batches and for results from individual specimens.
	8. Validate assay performance by using established analytical metrics such as accuracy, precision, coefficient of variation, sensitivity, specificity, linear range, limit of detection, and limit of quantification, as applicable.
	9. Establish acceptable reproducibility among technicians and participating laboratories and develop a quality assurance plan to ensure adherence to a detailed SOP and maintain reproducibility of test results during the clinical trial.
	10. Establish a turnaround time for test results that is within acceptable limits for use in real-time clinical settings.
Model development, specification, and preliminary performance evaluation	11. Evaluate data used in developing and validating the predictor model to check for accuracy, completeness, and outliers. Perform retrospective verification of the data quality if necessary.
	12. Assess the developmental data sets for technical artifacts (for example, effects of assay batch, specimen handling, assay instrument or platform, reagent, or operator), focusing particular attention on whether any artifacts could potentially influence the observed association between the omics profiles and clinical outcomes.
	13. Evaluate the appropriateness of the statistical methods used to build the predictor model and to assess its performance.
	14. Establish that the predictor algorithm, including all data pre-processing steps, cutpoints applied to continuous variables (if any), and methods for assigning confidence measures for predictions, are completely locked down (that is, fully specified) and identical to prior versions for which performance claims were made.
	15. Document sources of variation that affect the reproducibility of the final predictions, and provide an estimate of the overall variability along with verification that the prediction algorithm can be applied to one case at a time.
	16. Summarize the expected distribution of predictions in the patient population to which the predictor will be applied, including the distribution of any confidence metrics associated with the predictions.
	17. Review any studies reporting evaluations of the predictor?s performance to determine their relevance for the setting in which the predictor is being proposed for clinical use.
	18. Evaluate whether clinical validations of the predictor were analytically and statistically rigorous and unequivocally blinded.
	19. Search public sources, including literature and citation databases, journal correspondence, and retraction notices, to determine whether any questions have been raised about the data or methods used to develop the predictor or assess its performance, and ensure that all questions have been adequately addressed.
Clinical trial design	20. Provide a clear statement of the target patient population and intended clinical use of the predictor and ensure that the expected clinical benefit is sufficiently large to support its clinical utility.
	21. Determine whether the clinical utility of the omics test can be evaluated by using stored specimens from a completed clinical trial (that is, a prospective?retrospective study).
	22. If a new prospective clinical trial will be required, evaluate which aspects of the proposed predictor have undergone sufficiently rigorous validation to allow treatment decisions to be influenced by predictor results; where treatment assignments are randomized, provide justification for equipoise.
	23. Develop a clinical trial protocol that contains clearly stated objectives and methods and an analysis plan that includes justification of sample size; lock down and fully document all aspects of the omics test and establish analytical validation of the predictor.
	24. Establish a secure clinical database so that links among clinical data, omics data, and predictor results remain appropriately blinded, under the control of the study statistician.
	25. Include in the protocol the names of the primary individuals who are responsible for each aspect of the study.
Ethical, legal, and regulatory issues	26. Establish communication with the individuals, offices, and agencies that will oversee the ethical, legal, and regulatory issues that are relevant to the conduct of the trial.
	27. Ensure that the informed consent documents to be signed by study participants accurately describe the risks and potential benefits associated with use of the omics test and include provisions for banking of specimens, particularly to allow for ?bridging studies? to validate new or improved assays.
	28. Address any intellectual property issues regarding the use of the specimens, biomarkers, assays, and computer software used for calculation of the predictor.
	29. Ensure that the omics test is performed in a Clinical Laboratory Improvement Amendments-certified laboratory if the results will be used to determine treatment or will be reported to the patient or the patient?s physician at any time, even after the trial has ended or the patient is no longer participating in the study.
	30. Ensure that appropriate regulatory approvals have been obtained for investigational use of the omics test. If a prospective trial is planned in which the test will guide treatment, consider a pre-submission consultation with the US Food and Drug Administration.

This checklist applies to any clinical trial involving investigational use of an omics test that will influence the clinical management of patients in the trial, for example, the selection of therapy. In situations where an omics test will be evaluated retrospectively on valuable non-renewable specimens collected from patients who were prospectively enrolled in clinical studies, many of the checklist criteria are still applicable and the checklist can serve as a useful guide in judging the quality of the predictor development process and the strength and reliability of the evidence.

This paper is intended as an annotated companion to the short version of these guidelines published elsewhere [3]. Whereas that brief article provides a quick overview of the checklist, background on its development, and discussion of the context in which it is intended to be used, this longer paper elucidates the rationale underlying the development of the criteria in greater detail.

These are general guidelines, and it is recognized that there may be nuances in how they are applied to a particular omics test and clinical setting. The development of omics tests typically proceeds through a series of studies, and it may not be possible to address all of these criteria in early developmental studies. Ideally, investigators should consult this checklist during the research planning and test development phases so that critical evidence is systematically acquired and reported and, by the time the test is ready for definitive evaluation in a clinical study, the necessary evidence to comprehensively address the criteria has been obtained. It is hoped that researchers will find this checklist useful as they prepare background material for research proposals and clinical trial protocols.

### Specimen issues

1. Establish methods for specimen collection and processing and appropriate storage conditions to ensure the suitability of specimens for use with the omics test.

Numerous factors can alter a specimen?s molecular characteristics and influence the usability of a specimen, or analytes isolated from a specimen, for an omics assay [4-10]. The exquisite sensitivity of omics technologies to subtle biologic variations also implies that some of these technologies can be easily influenced by factors encountered in specimen collection, processing, and storage. Specimens or isolated analytes may become degraded or their omics profiles altered in unexpected ways. The entire life cycle of the specimen should be considered, beginning with the condition of the host when the specimen is first acquired (for example, patient has been fasting or is anesthetized) and including the procedure by which it is acquired (for example, surgical excision, core needle biopsy, venipuncture, bone marrow aspiration), the processing method (for example, snap-frozen in liquid nitrogen), use of stabilizers and preservatives (for example, ethylenediamine tetraacetate, neutral buffered formalin), and storage method (for example, -80?C, room temperature, vacuum sealed). Investigators should review all available data on these factors for the specimens used in the developmental studies for the omics test or should conduct further investigations to examine the influence of such factors on the omics test?s performance. It is important to document that the omics test will perform satisfactorily under the range of conditions in which the specimens will be obtained and stored in typical clinical settings; alternatively, more restrictive requirements for specimen collection, processing, and storage should be clearly specified before the test is used in a clinical trial or other clinical validation study.

2. Establish criteria for screening out inadequate or poor-quality specimens or analytes isolated from those specimens before performing assays.

In many situations it will be impossible to identify and record all of the factors that might influence the quality of specimens or isolated analytes. It may be necessary to make a decision about the acceptability of a specimen solely on the basis of the measurable characteristics of the specimen in hand. This requires that criteria for specimen quality be carefully specified in order to qualify a specimen or its isolated analytes as suitable for assay by the omics test. Such criteria might also be developed and used in addition to specifications on specimen acquisition, processing, and storage. Appropriate criteria will depend on the specimen type and the particular assay platform to be used. For omics assays based on RNA, the RNA Integrity Number (RIN; Agilent Technologies) is an example of such a quality metric [11], although thresholds for acceptable quality may be context dependent. The RIN is generally considered a valid quality criterion for RNA extracted from frozen specimens, but there is no consensus on its value for RNA extracted from formalin-fixed, paraffin-embedded specimens. Amount of DNA fragmentation might not be an important factor for immunohistochemical or fluorescence *in situ* hybridization assays applied to formalin-fixed, paraffin-embedded specimens, but omics assays might not perform well on highly fragmented DNA. In such cases, criteria assessing the extent of DNA fragmentation may be an essential component of the quality screen. A disadvantage of basing decisions for specimen acceptability only on the measurable characteristics of the specimen or isolated analytes is that this approach relies on those characteristics to capture the relevant influences of prior conditions.

3. Specify the minimum amount of specimen required.

The performance of an omics assay may be closely related to the amount (mass or volume) of specimen available or to the purity of the target material. In many cases, the composition of a specimen is a heterogeneous mix of cells of interest. For example, a surgical tissue specimen from a patient with cancer may consist of tumor cells admixed with necrotic tissue, normal epithelial cells, circulating blood cells, and stromal cells, and the presence of the non-tumor cells may dilute the target to be measured and cause false-negative assay results. Sometimes analyte enrichment techniques such as laser capture microdissection are used to enrich for cells of interest (for example, tumor cells) from whole-tissue specimens. Another common problem is extensive contamination of bone marrow aspirates with blood cells. Often, there may be a trade-off between specimen quality and quantity. For example, a partially degraded DNA sample might still be acceptable if the total amount is sufficient to contain an adequate number of high-quality DNA molecules. An extensive analysis of the suitability of DNA, RNA, and protein extracted from core needle biopsies of kidney cancer provides an example of how one might assess the quantity and quality of analytes that could be isolated from a particular type of specimen [12]. Criteria should be established not only for the starting amount of specimen, but also for the percent purity of the target cells or intact analyte of interest. These evaluations should be performed by individuals with appropriate expertise in histopathology.

4. Determine the feasibility of obtaining specimens that will yield the quantity and quality of isolated cells or analytes needed for successful assay performance in clinical settings.

Many omics tests are developed with retrospective collections of specimens that might have already been pre-selected to be of sufficient quality and quantity and thus may not be representative of the specimens that are likely to be obtained in the intended-use clinical setting. It might not be known whether this pre-selection has occurred. There may be no record of the number of patients for whom specimen collection was initially attempted or of the number of attempts that were made per patient until those attempts were either successful or aborted. Some specimens might have been previously collected in specialized research settings in which there were adequate expertise and resources to successfully execute the collections. Patients treated in the context of research studies may be more accepting of specimen collection and more tolerant of potentially invasive specimen collection procedures, leading to greater success in specimen collection in these settings than might be expected in routine clinical settings. To evaluate the feasibility of collecting the needed quantity and quality of specimens in a multicenter clinical trial or a routine clinical setting, it may be necessary to conduct a preliminary feasibility study in more realistic clinical settings. It is important to establish that the omics test is sufficiently robust and will perform acceptably for specimens likely to be encountered in clinical practice.

### Assay issues

5. Review all available information about the standard operating procedures (SOPs) used by the laboratories that performed the omics assays in the developmental studies, including information on technical protocol, reagents, analytical platform, assay scoring, and reporting method, to evaluate the comparability of the current assay to earlier versions and to establish the point at which all aspects of the omics test were definitively locked down for final validation.

The value of critically examining the history of an assay?s development before proceeding to a clinical trial is often underappreciated. Research laboratories in particular may modify their assay methods over time to improve assay performance or adapt to changing costs or availability of reagents, specimens, or instrumentation. Before an omics test is considered for use in a clinical trial, the assay methods used and the data gathered in the developmental stages should be carefully reviewed to determine whether the version of the assay underlying the omics test being proposed for the trial can be expected to generate data comparable to those generated by the former version(s) of the assay. This can be a particular challenge when using omics tests utilizing commercially available microarrays or other rapidly evolving technologies. This assessment should include not only the primary (?raw?) data generated by the assay but also any scoring method or interpretation rule (for example, positive versus negative) that is to be applied. If data generated in multiple laboratories were used in the development process, it should also be determined whether the different laboratories generated comparable data.

A fully specified assay method (see Criterion 6) is one of the critical aspects of a locked-down omics test. Additional requirements for a locked-down test include specific specimen requirements (see Criteria 1 to 3), detailed data pre-processing instructions, and a fully specified computational model for prediction (see Criteria 14 and 23). Before an omics test is used in a trial, it should have been validated in a pre-specified, locked-down form. If changes have been made to any aspect of the test, it must be established that the modified test produces results that are highly comparable to those of a version that has been previously validated clinically in locked-down form.

6. Establish a detailed SOP to conduct the assay, including technical protocol, instrumentation, reagents, scoring and reporting methods, calibrators and analytical standards, and controls.

The assay protocol should be sufficiently detailed to ensure its reproducibility. Elements in the SOP should be specific to minimize variation in the result when the assay is performed at different times, in different laboratories (if more than one laboratory will run the test), and by different technicians. The SOP should include not only the technical steps for conducting the assay, but also the instrumentation, reagents, scoring and reporting method, types of calibrators and analytical standards, controls, and quality control procedures for monitoring assay performance to ensure intra- and inter-laboratory reproducibility (see Criterion 7). To avoid ambiguity in usage of the terms ?calibrators?, ?standard?, and ?controls?, they are defined here as follows:

??A calibrator is a sample engineered to produce a specific value for a particular analyte and is used in the development of a calibration curve to standardize assay values from run to run.

??An analytical standard is a sample that has been extensively characterized and is expected to produce a consistent assay result in repeated assays over time.

??A control is a biological specimen that is available in sufficient quantity to include in multiple assay batches to monitor assay performance for potential drift; or a biological specimen that is expected to produce an unequivocally negative (negative control) or positive (positive control) result.

The SOP should be developed with attention to the expected context of use in the community after the conclusion of the clinical trial or confirmatory validation study. Parameters that are specific to reliability and feasibility for clinical use, like maximum turnaround time, should also be specified (see Criterion 10). To ensure that the test results are clearly transmitted for appropriate clinical interpretation, the SOP should also exactly specify the format in which the output of the assay will be reported.

7. Establish acceptability criteria for the quality of assay batches and for results from individual specimens.

Unexpected technical problems can occur during the course of an assay, producing aberrant results for individual samples or entire assay batches. A quality monitoring system should be in place to detect these problems. For example, a comprehensive set of performance metrics has been proposed for liquid chromatography?tandem mass spectrometry systems in proteomics analyses [13]. The acceptability criteria may focus on bias or precision or both. Control samples, analytical standards, and blinded replicate samples, when used consistently, can be effective tools for detecting assay problems and ensuring consistency of results [14-16]. The recommended types of controls, analytical standards, and replicates, along with the criteria used to determine acceptability of assay results, should be specified in the assay protocol. Routinely measuring individual samples in duplicate or triplicate can also be helpful to identify unreliable readings, though it might not be helpful to detect biased readings. Lastly, these procedures should also include replicate analyses of controls and standards over time to ensure the temporal stability of the omics assays and isolated analytes under the specified conditions of storage. This issue is of particular importance in the context of prospective trials in which patient accrual and sample collection and analysis will occur over a protracted period of time. The use of these types of quality assurance and quality control procedures will increase confidence in the assay results and help to guard against serious assay failures that could affect patient care directed by the test results.

8. Validate assay performance by using established analytical metrics such as accuracy, precision, coefficient of variation, sensitivity, specificity, linear range, limit of detection, and limit of quantification, as applicable.

Before an omics test is used in a clinical trial, the analytical performance of the assay should be evaluated to establish the assay?s analytical validity. This evaluation should examine performance metrics, such as accuracy, precision, coefficient of variation, sensitivity, specificity, linear range, limit of detection, and limit of quantitation, as applicable for the particular test under study. A study reported by Tabb *et al*. [17] provides examples of the types of repeatability and reproducibility assessments that could be made in proteomic identifications by liquid chromatography?tandem mass spectrometry. A number of helpful guidance documents and templates that outline best practices for characterizing assay analytical performance are available [18-27].

If individual biomarker measurements are combined (for example, as a weighted average), it may be useful to understand the analytical performance characteristics of the individual biomarker measurements that enter into the omics prediction model, particularly in the model development stage. However, assessment of the analytical characteristics of the final result produced by the omics test is of primary importance as the test is moved into use in a clinical trial. The bias or imprecision in the final result will have a direct impact on the patient care that is guided by the test. For example, a genomics test might produce a continuous risk score, calculated as a linear combination of multiple biomarker measurements. The reproducibility of the final risk score generated by such a linear predictor will depend on the bias and precision of the measurements of the individual biomarkers and the weight given to each biomarker in the risk score. A cutpoint may be applied to the risk score for translation into a clinical classification. The reproducibility of the final clinical classification will then depend on the reliability of the risk score and the proportion of the risk scores that cluster near the cutpoint. Higher variability can be tolerated in a risk score when it is far away from a cutpoint, because the final clinical classification is unlikely to change due to inaccuracy in the risk score. Special considerations apply to prediction models that require complex iterative or stochastic calculations for evaluation, in contrast to the simple setting of the linear risk score just discussed (see Criteria 15 and 16).

Assessment of the analytical performance of an omics test should be carried out with a set of clinical specimens that reflect the expected range of combinations of component omics variable values. The clinical specimens used in analytical validation studies should be representative of the types of specimens anticipated in the targeted clinical setting, and the patients from whom those specimens are collected should cover a spectrum similar to that of patients for whom the test is intended.

9. Establish acceptable reproducibility among technicians and participating laboratories and develop a quality assurance plan to ensure adherence to a detailed SOP and maintain reproducibility of test results during the clinical trial.

Unexpectedly large differences in assay results can be caused by the particular technician performing any step of an assay, as well as by differences in environments or standard practices across laboratories or clinical sites. Strict adherence to detailed SOPs for specimen collection, processing, and handling and for assay procedures can substantially reduce the amount of variation in omics test results due to these factors; however, it may not be possible to completely eliminate this variation [28-31]. Preliminary data should be presented to establish acceptable reproducibility across technicians, analytical instruments or platforms, laboratories, and clinical sites. Because laboratory and clinical staff and environments can change and new clinical sites may be added over time, it is also important to have in place quality assurance programs and quality monitoring processes to ensure that comparability is maintained throughout the course of a validation study or clinical trial. Recommended procedures might include initial training and qualification of staff, periodic refresher training sessions, and use of blinded replicate or control specimens to directly assess the comparability of assay results over time and across laboratories. If the test is to be used to determine treatment, it must be performed in a Clinical Laboratory Improvement Amendments (CLIA)-certified laboratory. A CLIA-certified laboratory would typically have these procedures already in place, although CLIA certification would not ensure comparability across laboratories when more than one laboratory is involved in performing assays for a study.

10. Establish a turnaround time for test results that is within acceptable limits for use in real-time clinical settings.

For an omics-based test to be useful in clinical practice, it must be feasible to collect and process the required specimen, complete the assay, generate and confirm the validity of the primary data, compute the predictions, and have the result available within an acceptable time frame without substantially delaying the usual timing of clinical decisions regarding treatment or other follow-up care. Because many omics-based tests are developed and preliminarily validated on retrospective specimen collections, in some cases there is no prior opportunity to assess the feasibility of using the test in real time. Feasibility studies should be conducted prior to the initiation of a trial to ensure that the necessary infrastructure and resources will be in place to collect and process the required specimens according to the specified methods, that there will be sufficient capacity in the laboratories performing the assays, and that it will be possible to achieve timely data submission and processing to generate the predictions for individual patients in the trial.

### Model development, specification, and preliminary performance evaluation

11. Evaluate data used in developing and validating the predictor model to check for accuracy, completeness, and outliers. Perform retrospective verification of the data quality if necessary.

It is strongly advised that a critical and independent evaluation be conducted of the quality of the data used to develop and preliminarily validate an omics predictor. Unlike data for clinical trials, which are typically collected under standardized and carefully quality-controlled conditions, in many cases data used to develop predictors are based on assays conducted on banked clinical specimens for which clinical and pathologic data may have been assembled from retrospective record reviews. Both the omics assay data and the clinical and pathologic data used in the studies should be carefully reviewed for any evidence of errors, inconsistencies, or biases resulting from careless or incomplete data collection and clinical annotation.

In some cases the omics assays might have been conducted by others and only the omics data, and not the specimens, are available. In these situations there may be little information available about the quality of the specimens or assay procedures used. Quality metrics have been proposed for data from some types of omics assays [32-35], and these can be helpful to identify potential problems. Quality assessments should be performed on the primary omics data from original sources, if available, as well as on any pre-processed data to confirm that no errors were introduced during data handling and processing.

Some omics data problems can be identified by use of simple descriptive statistics. Unusually high correlation between molecular profiles of two different specimens may indicate an unintended duplication of specimen labels. When analyzing data merged from several different studies, one should always assess for high correlations between specimens that could occur if there is overlap in the patients whose specimens were examined in the different studies. For nucleic acid-based assays, cross-contamination of specimens can occur and distort genetic variant profiles. It is worthwhile to conduct these preliminary data analyses to allow for identification and removal of problematic data and increase confidence in the data?s reliability.

Clinical and pathologic data should be examined for evidence of internal inconsistencies (for example, recurrence date after date of death), extreme observations (for example, 9-year-old patient with breast cancer) or implausible data combinations (for example, male patient with ovarian cancer). Extreme observations or unusual data combinations can have a large influence on the form of a predictor or its performance and therefore should be subject to verification when possible. Although it will not always be feasible to determine whether unusual-looking data are erroneous, at the least the impact of any suspect data on the model or its performance should be assessed.

12. Assess the developmental data sets for technical artifacts (for example, effects of assay batch, specimen handling, assay instrument or platform, reagent, or operator), focusing particular attention on whether any artifacts could potentially influence the observed association between the omics profiles and clinical outcomes.

Some features of omics profiles can arise from artifacts introduced due to variations in specimen handling, assay reagents, or instrumentation [36]. It is important to check the omics data for evidence of these artifacts. Methods of specimen handling or processing can change over time or differ across clinical sites. Over time, laboratory instruments can drift, reagent lots can change, and assay results can exhibit distinctive ?batch effects? due to changes in technique, environmental conditions, or operators. Technology platforms (instrumentation, software, and reagents) can become obsolete, requiring replacement with new versions. A laboratory information management system can be useful for tracking some of these factors to allow for detection of possible problems and troubleshooting. Although attempts can be made to correct for these artifacts through data adjustment and/or use of replicated assays of analytical standards or calibrators, such adjustments often do not completely remove them. The residual effects of these artifacts introduce ?noise? into the data and may degrade the performance of the omics predictor.

The best line of defense against technical artifacts in the development stage of an omics predictor is quality monitoring and good experimental design to avoid confounding technical factors with important biological effects or clinical outcomes [37]. For example, if specimens from patient responders and non-responders were assayed in separate batches, an omics predictor developed from those data might predict only assay batch, not clinical outcome. This can be avoided by randomly assigning specimens to assay batches. Other forms of confounding can be more subtle. If patients accrued at one clinical site tend to have worse prognoses than those accrued at a second clinical site, and the two sites process specimens differently in ways that affect the omics profile, an omics predictor developed from such data could end up predicting the specimen processing method and have little true value for predicting clinical outcome. Whenever possible, SOPs for specimen handling should be put in place across all clinical sites to minimize these nuisance effects and avoid confounding specimen handling with patient prognostic characteristics that vary by clinical site. If it is not possible to standardize procedures, or if existing collections of specimens are being used (potentially accrued from multiple clinical sites), it is important to demonstrate, perhaps through multivariable statistical analyses, that the omics predictor has the ability to predict outcome within each clinical site and after adjustment for other standard clinical or pathological variables.

Artifacts and confounding factors are frequently encountered in omics studies because it is often necessary to pool across multiple clinical sites or multiple data sets to amass a sufficiently large set of cases with omics data and clinical and pathologic data with which to build or preliminarily validate an omics predictor. Investigators who first collect specimens and perform omics assays on those specimens should make concerted attempts to record information about specimen handling and ancillary assay variables such as assay batch identifiers so that statistical analyses can be performed to examine their influence on the omics profiles. Unfortunately, this information is unavailable for many retrospective data sets. However, it can sometimes be partially recovered if primary omics data files produced by software systems packaged with the assay platforms are available. Depending on the omics assay platform, some of this information may be embedded in the primary data files. For example, the header lines in Affymetrix GeneChip CEL files contain the date and often information about the laboratory or operator. The potential for these unknown nuisance factors to affect the performance of an omics predictor underscores the need for external validation of omics predictors before they are used to guide patient care.

13. Evaluate the appropriateness of the statistical methods used to build the predictor model and to assess its performance.

The high dimensionality of omics data and the complexity of many algorithms used to develop omics predictors present many potential pitfalls if proper statistical modeling and evaluation approaches are not used. Various statistical methods and machine learning algorithms are available to develop models, and each has its strengths and weaknesses [38,39]. There is no uniformly best algorithm for developing a predictor model [40]. One of the earliest studies to compare several methods for development of predictors from gene expression microarray data showed that simple linear diagonal discriminant analysis and nearest-neighbor methods performed as well or better than those developed with a variety of more complex approaches on multiple data sets [41]. A subsequent study conducted by the MicroArray Quality Control Consortium [42] compared predictors developed by 36 independent teams analyzing six microarray data sets to develop predictors for 13 cancer and toxicology endpoints. That study concluded that the performance of the predictors that were developed ?depended largely on the endpoint and team proficiency and that different approaches generated models of similar performance.? More complex modeling approaches, especially those involving regularization (approaches to constrain complexity of models) and optimized feature selection [43], theoretically have the potential to produce better-performing predictors. However, they are sophisticated tools that need to be applied by skilled hands. In situations where the number of omics variables far exceeds the number of independent subjects or specimens, current evidence from comparative studies has not convincingly demonstrated the advantage of highly complex modeling approaches.

A pervasive problem in the omics literature is that algorithms to develop predictors are often applied naively, and flawed approaches are used to assess the predictor?s performance [40,44,45]. The more complex the algorithm, the greater the chance that it will be misunderstood or applied incorrectly in inexperienced hands. The two most common pitfalls in developing predictors and assessing their performance are model overfitting and failure to maintain strict separation between the data used to build the predictor model and the data used to assess the predictor performance. These two pitfalls occur in a large number of published papers claiming to have developed omics predictors with good performance [44,45]. The result is failure of many omics predictors when they are tested on a truly independent data set [46,47].

Overfitting occurs when a statistical model describes random error or noise instead of the underlying relationship. Modeling strategies that allow for extremely complicated relationships between one or more independent variables and the dependent (outcome) variable and models that are built from very large numbers of variables are particularly prone to overfitting because they can exaggerate minor fluctuations in the data [40]. For omics data, the number of variables that are available to build a predictor typically greatly exceeds the number of independent subjects for whom omics profiling data have been obtained; therefore, the potential to overfit models to high-dimensional omics data can be enormous. For studies in which an omics predictor is developed for a time-to-event endpoint such as survival, the number of observed events (for example, deaths) is a major determinant of the reliability of the fitted model. For diagnostic studies aiming to build a predictor for disease state or class (for example, cancer or no cancer), the number of patients in the least prevalent class most strongly affects the ability to develop a reliable diagnostic model. Modeling approaches that include regularization to constrain the complexity of the model and limit the influence of individual variables or observations are particularly helpful in reducing the potential for overfitting. When selecting among candidate models, it is important to use appropriate procedures to assess model performance to guard against incorrectly selecting an overfitted model. Overfitted models will generally have poor predictive performance on new data sets.

 Validation of model performance on a completely independent external data set is optimal, but there can be ambiguity in the required degree of independence. For the strongest independent external validation, the specimens should be collected under the intended conditions at a different site, and the assays should be conducted according to the final SOP in a different laboratory and by different laboratory personnel. If sufficient numbers of patients or specimen sets are available, a series of validations might be performed in which an additional factor is varied in each successive validation attempt to systematically establish robustness of the omics predictor to these conditions. It is important to clearly describe the conditions under which each validation is attempted so that the strength of each validation can be evaluated in the proper context.

It is not always possible to obtain external data collected under the exact target clinical setting and meeting all of the quality standards for the most rigorous type of validation. In some cases, there might be an external data set, but the specimens or assay protocols might not exactly match those intended for the omics test; in other instances, the clinical setting might be outdated, for example, because standard treatment practices have changed. In these circumstances, one must rely on use of model development techniques designed to avoid overfitting, and one can only make assessments of performance internal to the data set used to build the model.

Performance assessments based on re-use of data used in model building are informative only when appropriate internal validation strategies are used. Despite standardization of assays and attempts to avoid model overfitting, a predictor model will nearly always fit better to the data used to develop it than it does to completely independent data. For models built from high-dimensional omics data, simply ?plugging in? to a predictor exactly the same data that were used to build it in order to estimate the predictor?s performance ? a so-called resubstitution estimate ? results in a highly optimistically biased estimate of performance [40,45]. Resubstitution estimates of performance for predictors built from high-dimensional omics data are uninterpretable and should not be reported. Unfortunately, resubstitution estimates of model performance can still be found in some published articles. Appropriate alternatives to the na?ve resubstitution method are available and should be used for internal validation.

The guiding principle for how to avoid optimistically biased estimates of predictor performance is to never estimate a predictor?s performance by using data that were used to derive the prediction model. The easiest way to ensure separation of model building and assessment is to have completely independent training and validation data sets so that an external validation can be performed. For the most rigorous external validation:

??The predictor to be tested must be completely locked down and there must be a pre-specified performance metric. The lockdown includes all steps in the data pre-processing and prediction algorithm.

??The independent validation data should be generated from specimens collected at a different time, or in a different place, and according to the pre-specified collection protocol.

??Assays for the validation specimen set should be run at a different time or in a different laboratory but according to the identical assay protocol as was used for the training set.

??The individuals developing the predictor must remain completely blinded to the validation data.

??The validation data should not be changed based on the performance of the predictor.

??The predictor should not be adjusted after its performance has been observed on any part of the validation data. Otherwise, the validation is compromised and a new validation may be required.

Internal validation can be used when no external validation set is available and is also helpful to use during the model building stage to generate preliminary estimates of model performance to monitor and guard against overfitting. Either the original data set can be split into ?training? and ?testing? subsets [48] or a variety of data resampling techniques can be used to iteratively build the predictor model on a portion of the data and test it on the remaining portion.

Split-sample validation, in which a single data set is split into two parts, has the advantage of being computationally simple. It also provides the flexibility needed to make subjective decisions in the model building process on the training portion of the data. As a further check on model performance in split-sample validation, one can interchange the training and testing subsets to evaluate whether the models built using each subset have similar performance on the other subset [42]. Sometimes what is viewed as a single data set may actually be an amalgamation of several smaller sets. Different portions of the data might represent omics data that were generated in different laboratories or from specimens collected at different clinical sites. Even if all laboratories and clinical sites followed a common protocol for specimen collection and omics assays, subtle differences can arise. In this situation, the most challenging type of split-sample validation, and most representative of ?real world?, is to partition the data into training and testing sets to minimize the overlap in the laboratories or clinical sites represented in the two sets. A disadvantage of split-sample validation is its inefficiency. This is because only a fraction of the data is ever used to build the predictor model. Split-sample validation applied to small or moderate size data sets tends to yield biased results because it underestimates the performance of a model that could be built using the entire data set.

Resampling methods have an advantage over split-sample validation in that they use the full data set but iteratively select which portion of the data serves as a training set, so that at the end of the iterations, each case has been in at least one training subset and in at least one validation subset. Examples of resampling methods include various forms of cross-validation (for example, leave-one-out, k-fold where typically k = 5 or 10) and bootstrapping. The best choice of resampling method for a particular problem depends on the size of the data set and the desired trade-off between bias in the performance estimate and variance of the performance estimate [49].

Internal validation has two major limitations. First, if the cases selected for study are not representative of the intended-use population or there are technical artifacts affecting the entire data set, any subsets of the data will inherit those same problems. This is especially of concern when the bias is from technical artifacts that confound the association between the omics profiles and the clinical endpoint of interest (for example, specimens from patients with favorable clinical outcomes run in a different assay batch than those from patients with unfavorable clinical outcomes). This can lead to biased estimates of test performance that cannot be detected unless the test is evaluated on a completely independent data set not subject to these same problems. Second, iterative resampling methods are applicable only if the process used to build the predictor model is entirely algorithmic and requires no subjective judgment. Subjective judgment can potentially and subtly enter into several aspects of building predictor models. These aspects may include decisions about constraints on the number of variables in the model, constraints on the weight given to any single variable, how to handle unusual measurements, how to summarize redundant variables, and where to set cutpoints on risk scores for clinical decision making. Although many of these aspects can be decided in a fully algorithmic fashion, many investigators are reluctant to rely on completely automated model building methods.

The principle of separation of training and validation sets can be violated in several more subtle ways. Sometimes investigators use a split-sample approach and develop a predictor model using only a portion of the data, but then present the model performance estimates on the combined training and validation sets rather than on the validation set only, as would be appropriate. This still leads to an optimistic bias in the performance assessment because the resulting performance estimate is a hybrid of an optimistically biased resubstitution estimate on the training data and an independent estimate on the validation data [50]. Another common error made when applying iterative resampling validation approaches is to perform an initial screen for omics variables using the entire data set to identify those variables that are univariately most informative for predicting the clinical outcome of interest, and then to perform iterative resampling to fit the predictor model using only that subset of selected variables. The leak of information resulting from that initial screening on the full data set to reduce the number of variables can be substantial and can lead to optimistic bias in performance estimates nearly as large as for resubstitution estimates [40,51].

Other practices that can introduce bias into the reported performance of a predictor model include selective inclusion or exclusion of certain cases in either the training or the validation sets to obtain improved estimates of predictor performance, testing performance in multiple patient subgroups within the full cohort, and trying multiple model building strategies on the training set, but then reporting only the one that yields the best performance estimate on the validation set. All of these practices are examples of multiple testing, and they can lead to spurious findings and optimistically biased estimates of reported predictor performance.

Regrettably, numerous papers published in reputable journals reporting to have developed omics predictors with good performance have used flawed methods of evaluating predictor performance such as those just discussed. Thus, publication in a peer-reviewed journal should not be taken as assurance that the performance of an omics predictor has been confirmed. Published articles should be critically reviewed for soundness of the methods used. If sufficient information about the methods is not provided in the published article, it may be necessary for this information to be obtained directly from the predictor developers, along with any primary data and computer code that are needed to independently reproduce the predictor development and validation results before the omics predictor is used to direct therapy in a clinical trial.

14. Establish that the predictor algorithm, including all data pre-processing steps, cutpoints applied to continuous variables (if any), and methods for assigning confidence measures for predictions, are completely locked down (that is, fully specified) and identical to prior versions for which performance claims were made.

A multitude of steps occur from the point at which primary omics data are generated from a specimen until a final result is produced from the omics predictor. These include data pre-processing steps such as overall data quality assessments, exclusion of unreliable measurements, data normalization, calculation of intermediate summary statistics (for example, calculation of gene-level summary expression levels from probe intensity values in microarray data), calculation of a score (possibly subjected to a cutpoint for clinical decision making), or prediction of an outcome. A standardized format for reporting the test result should be developed to ensure proper clinical interpretation. Elements of the report might include a continuous score (for example, probability of disease recurrence) or discrete classification (for example, disease subtype) or both, perhaps accompanied by some measure of confidence or statistical uncertainty interval for the result (for example, strong positive, equivocal, ?uncertainty in risk score is ?10%?) and a recommended clinical action (for example, consider adjuvant chemotherapy, contraindication for a drug class).

Before an omics predictor is used in a clinical setting where it will influence patient care, all aspects of the data processing and prediction calculation and reporting should be recorded in a detailed, dated SOP document and should remain unchanged from their form when the predictor forming the basis for the clinical test was locked down for the final validation. Changes to any of these steps, including adjustments to cutpoints applied to continuous risk scores, can alter the performance characteristics of a predictor. Such changes may necessitate a new validation of the predictor before it is used to direct patient care unless other results, for example, from an assay bridging study, can provide convincing evidence that the performance of the predictor has not been adversely affected by the changes. Several reproducible research software and data management systems that can be helpful to document the process of building and validating predictors are now available (for example, Accessible Reproducible Research [52], GenePattern [53], Sweave [54], knitr [55], markdown with Rstudio [56], git [57]). To achieve complete transparency in the omics test development process, raw data should be made available for review, and the data sources should be identified. Evaluations performed to assess data quality and to check for technical artifacts should be reported. The computational environment (languages, software versions, libraries, hardware, and cloudware) should be identified, and programmatic scripts used in data pre-processing (for example, normalization) and model development and testing should also be available for review. Public access to all of the above is desirable, but at minimum, these items must be available for review by the sponsors and relevant oversight bodies for the proposed trials in which the omics predictor is to be used.

15. Document sources of variation that affect the reproducibility of the final predictions, and provide an estimate of the overall variability along with verification that the prediction algorithm can be applied to one case at a time.

The association between an omics test result and the clinical outcome the test is intended to predict will be attenuated if the testing process lacks reproducibility. Test results obtained for a given individual can vary for numerous reasons, including biological heterogeneity of the specimen (for example, distinct clonal subpopulations of cells or necrotic regions in a tumor), variation in specimen handling, technical variation in the assay, and numerical variation in the prediction algorithm. Some of these sources of variation can be controlled and others cannot. Biological heterogeneity within a specimen cannot be controlled, but it must be understood. If there is substantial biological heterogeneity and it cannot be determined that one portion or region of the specimen provides the omics information of most relevance to the clinical outcome (for example, the leading edge of the tumor, the area of the tumor with highest grade, or the stem cell compartment), then it is unlikely that the omics test will produce clinically reproducible and informative results. Variation due to specimen handling and assay technical variation is best controlled through careful specification of SOPs and quality monitoring. Numerical variation in the prediction algorithm can be controlled through choice of the algorithm or algorithm settings. Multiple reproducibility assessments may be required to fully understand the relative contributions of all of these sources of variation to the overall variation in the predictions that could be obtained for a given individual.

Reproducibility assessments should be reported in sufficient detail to allow others to understand the sources of variation that are being evaluated. For example, two separate portions of a tumor that are independently subjected to the analyte extraction process (for example, mRNA extraction from a tumor specimen), assay procedure (for example, gene expression microarray analysis), and prediction algorithm would be expected to exhibit more variability than replicate assays of a single sample of extracted analyte that is split and run through the omics assay process and prediction algorithm. Studies should be conducted to evaluate the robustness of omics test results to variations in specimen collection, processing, and storage if tight controls on these factors are not specified as part of the SOPs for the testing process. Specimens used in reproducibility studies should be comparable to the clinical samples for which use of the omics test is being proposed. Because more highly reproducible results are likely to be obtained on cell lines or artificially derived specimens that have been carefully prepared in a laboratory setting than on actual clinical samples that were collected under less predictable conditions, variability assessments made on cell lines or other derived specimens are likely to substantially underestimate the variability that could be experienced in clinical practice.

Variation in predictions due to the numerical algorithm that mathematically evaluates the model is the most straightforward to assess. This variation can be assessed independently of the biological and assay technical variation, but it will still contribute to the overall variation in the predictions for any given individual. This numerical variation arises only for prediction models that cannot be evaluated as a simple formula and require evaluation by an iterative or stochastic computerized algorithm. A stable computer algorithm should produce highly similar results when exactly the same primary omics data are used on independent occasions as the model input. Often computerized algorithms are needed because evaluation of the prediction model involves complex mathematical equations that can be solved only by the use of iterative numerical approximations. For example, Markov Chain Monte Carlo methods are iterative mathematical algorithms often used in Bayesian statistical modeling approaches [58]. Other prediction modeling approaches require a computer algorithm because they involve combining many models or decision algorithms where each component model or algorithm is built using only a randomly selected subset of the patient data (for example, random forests [59,60]) or using only a small subset of randomly chosen variables among a much larger number of variables available (for example, shotgun stochastic search [61]). In these situations, any variability associated with the iterative calculation or random-component model selection is incorporated into the variability of the final predictions. Depending on the particular algorithm used and the data set to which the algorithm is applied, it is possible that the randomness introduced by the computerized algorithm could be substantial. This variability becomes part of the variability in the test result and must be assessed along with the assay analytical performance (see Criterion 8). Sometimes such instability can be addressed through measures such as locking down random number seeds used by iterative numerical algorithms or by saving information about the exact component models that are combined into a final model. If the numerical variability in the final results cannot be adequately controlled, then these complex models will not be suitable for making clinical predictions where it is expected that the same set of observed omics variables should lead to a consistent final test result.

Many studies in which omics predictors have been developed have used data pre-processing methods that induce interdependencies of pre-processed data or predictions made on a collection of specimens. One circumstance in which this occurs is when the pre-processing or prediction algorithm that is applied to an individual specimen depends on the other specimens that happen to be processed with it. A simple example of when such a dependency could occur is when each measured variable is centered by subtracting that variable?s mean value calculated across a collection of specimens. A more complex example is the widely used Robust Multi-array Average method for calculating gene-level summaries from probe sets in the Affymetrix GeneChip system [62,63]. The Robust Multi-array Average method incorporates data from a collection of microarrays to fit a model that includes terms to estimate probe-specific sensitivities, and it is used to produce the gene-level summaries for each probe set on an Affymetrix GeneChip. For implementation in a clinical trial in which individual patients will be accrued to the trial over time, methods that require group processing of omics profiles must be modified to allow for processing one omics profile at a time. Some investigators have addressed this issue by using a fixed reference set of omics profiles to which each new omics profile is added and then removed for purposes of pre-processing data [64-66].

Some early methods of identifying tumor subtypes, such as intrinsic breast cancer subtypes [67,68] or subtypes of diffuse large B-cell lymphomas [69], used clustering methods. These clusters were found to have prognostic significance in addition to biological significance, but in order to be applicable to the classification of single specimens from individual patients, prediction algorithms had to be developed. Clustering algorithms used to identify subtypes may produce results that are sensitive to data pre-processing methods such as variable centering and may be dependent on the characteristics of the collection of specimens that are processed together [70]. Therefore, a predictor model has to be developed to reliably identify the subtypes without having to use clustering methods or other approaches that require processing a collection of specimens in order to make a prediction for a single case. For example, a risk prediction model was developed into a clinical test that could make a diagnosis of an intrinsic breast cancer subtype for a single patient tumor sample [71]. The basic principle is that one should be able to perform all the steps of the omics assay and prediction algorithm to produce a result for a given specimen without regard to other specimens that are being evaluated at the same time.

16. Summarize the expected distribution of predictions in the patient population to which the predictor will be applied, including the distribution of any confidence metrics associated with the predictions.

Many omics predictors are developed on existing data sets or on retrospective collections of specimens. The cases included in those studies do not necessarily comprise a representative sample of the clinical population for which the omics test is intended, or from any well-defined population. Sometimes cases are intentionally selected to overrepresent extremes of clinical outcome (for example, very short- or very long-term survivors). In other circumstances, the collection of cases studied are non-representative due to other practical constraints, such as ability to obtain consent, vital status of the patient, or amount of available specimen. In addition, some prediction algorithms produce a confidence metric associated with each prediction, which might relate, for example, to the proximity of a prediction risk score to a previously defined cutpoint for clinical classification. If the patients whose specimens were used in the developmental studies were not representative of the intended-use population, the distribution of the predictions and their associated confidence metrics in the developmental studies might not be representative of the distribution expected when the test is applied in clinical practice. If the predictions in the intended-use population are highly skewed toward predicting one clinical outcome over another, or if many predictions have low associated confidence, the predictor may not have a sufficiently large clinical impact to be useful. Therefore, assessment of these distributions should play a role in the decision about whether to pursue the clinical development of an omics predictor.

17. Review any studies reporting evaluations of the predictor?s performance to determine their relevance for the setting in which the predictor is being proposed for clinical use.

Many studies report to have performed validations of omics predictors, but the term ?validation? is used in many different ways. A variety of questions should be asked to assess the strength and relevance of any study that claims to have validated an omics predictor. Sometimes a technical validation has been performed to show that an alternative assay methodology produces measurements that have significant correlation with the originally measured omics variables. Although technical validations provide some assurance that the study results are not wholly artifacts of the assay process, they do not provide any clinically relevant validation. Other types of validations in preclinical systems, for example, drug sensitivity experiments in cell lines, may support biological plausibility, but they do not provide clinical validation. For a study to provide a clinical validation, there must be a predefined and clinically meaningful performance metric for the predictor, and the clinical setting (for example, disease type and stage, specimen format) must be similar to the intended-use setting.

There are well-established criteria for evaluating the performance of models used to predict risk [72-74], and guidelines have been developed for informative reporting of studies on the prediction of genetic risk [75] and on prognostic [76,77] and diagnostic [78,79] markers. These criteria and guidelines are applicable to a wide variety of omics predictors. The specific choice of performance metric (for example, sensitivity and specificity, positive and negative predictive value, C-index, area under the receiver operating characteristic curve) [72-74] and the benchmark performance level that must be attained will be dependent on the intended clinical use. An omics test intended for screening a large healthy population for disease (for example, a serum proteomic screening test for ovarian cancer) must have very high specificity and positive predictive value, and its predictive value must be calculated by using a disease prevalence that is appropriate for the intended screening population [80]. A test intended to predict the risk of disease recurrence to guide decisions about additional therapy (for example, a recurrence risk score to identify patients with early-stage breast cancer who do or do not need adjuvant chemotherapy) should have high sensitivity to identify patients in whom disease will recur. Demonstration that a predictor?s output is statistically significantly associated with the clinical endpoint it aims to predict is not sufficient evidence of acceptable performance for clinical use [81].

18. Evaluate whether clinical validations of the predictor were analytically and statistically rigorous and unequivocally blinded.

Unequivocal evidence of rigorous validation is required whenever a predictor is proposed for use in a clinical trial where it will influence patient care. Ideally, this should be a blinded external validation on a completely independent specimen set. Requirements for a rigorous blinded external validation include signed and dated documentation that the predictor was fully specified in locked-down form prior to the release of any validation data. This documentation must include an SOP for all aspects of the omics assay, including data processing steps and the prediction algorithm. A mechanism for blinding of clinical outcome data should have been in place, for example, under control of a third-party ?honest broker? or an independent statistics and data center that maintains data in a secure system that complies with US Food and Drug Administration (FDA) guidelines for the maintenance of clinical data in computerized systems [82]. If an external validation is not possible because, for example, no appropriate independent specimen set is available, then existing results from internal validations must be carefully evaluated to determine whether the internal validations were rigorous enough to provide reasonable confidence in the predictor?s performance. Findings of any validation attempts should be reported regardless of whether the results were favorable or unfavorable.

19. Search public sources, including literature and citation databases, journal correspondence, and retraction notices, to determine whether any questions have been raised about the data or methods used to develop the predictor or assess its performance, and ensure that all questions have been adequately addressed.

Omics research has been at the forefront of efforts to promote the public availability of data, and there has been unprecedented sharing of computational algorithms and computer code. Without this sharing of data and algorithms, the sheer volume of data and the complexity of many analyses conducted with omics data would have made it virtually impossible to reproduce many omics study results. On occasion, questions arise about data or analytic approaches when others try to reproduce results using publicly available data, methods, or computer code. Whenever an omics predictor is to be used in a trial or other clinical setting where it will influence patient care, there must be transparency so that any concerns about accuracy of data or appropriateness of methods can be promptly and fully addressed before resources are expended to pursue further development of the predictor, and certainly before the predictor is used clinically.

### Clinical trial design

20. Provide a clear statement of the target patient population and intended clinical use of the predictor and ensure that the expected clinical benefit is sufficiently large to support its clinical utility.

Many published omics studies report statistically significant associations between omics predictor results and clinical endpoints. Although the presence of such an association may establish the clinical validity of the test, statistical significance (for example, *P* >0.05) does not always translate into a clinically meaningful association or provide clinically useful information. Unless the omics predictor provides new information that is readily interpretable and useful to the physician and patient in making treatment decisions, the investment of resources in developing a clinical test may be wasted. To establish clinical utility, as opposed to clinical validity, there must be evidence suggesting that use of the test is likely to lead to a clinically meaningful benefit to the patient beyond that provided by current standards of care [83,84].

Design of a clinical trial for definitive evaluation of an omics test must begin with a clear statement of the target population and the intended clinical use. Information about the anticipated distribution of test results in the population and the magnitude of the expected effect or benefit from use of the test should be gathered from preclinical or retrospective studies. On the basis of that information, it should be determined whether it will be feasible to design a trial or clinical study of sufficient size to demonstrate clinical utility. Ideally, the size of the expected benefit from use of the omics test will have been estimated from multivariable analyses as being beyond that provided by knowledge of standard clinical or pathologic factors.

21. Determine whether the clinical utility of the omics test can be evaluated by using stored specimens from a completed clinical trial (that is, a prospective?retrospective study).

In some instances, a candidate prognostic or predictive omics test for an existing therapy can be evaluated efficiently by using a prospective-retrospective design, in which the omics test is applied to archived specimens from a completed trial and the results are compared with outcome data that have already been collected [85]. The retrospective aspect of this design requires that the assay can in fact be performed reliably on stored specimens. The ?prospective? aspect of the design refers to the care taken at the outset of the trial to ensure the following:

??The patients in the trial are representative of the target patient population expected to benefit from the omics test.

??There is a pre-specified statistical analysis plan.

??Sufficient specimens are available from cases that are representative of the trial cohort and intended-use population to fulfill the sample size requirements of the pre-specified statistical plan, and those specimens have been collected and processed under conditions consistent with the intended-use setting.

In general, two such prospective-retrospective studies producing similar results will be required to have confidence that the clinical utility of the test has been established.

22. If a new prospective clinical trial will be required, evaluate which aspects of the proposed predictor have undergone sufficiently rigorous validation to allow treatment decisions to be influenced by predictor results; where treatment assignments are randomized, provide justification for equipoise.

A variety of designs have been proposed for phase III clinical trials incorporating biomarkers [86-88]. There are three basic phase III design options that are frequently considered for assessing the ability of a biomarker to identify a subgroup of patients who will benefit from a new therapy. These are the enrichment design, the stratified design, and the strategy design. In the enrichment design, only patients who are positive for the biomarker are randomized to the standard or new therapy. This approach can answer the question of whether biomarker-positive patients benefit from the new therapy, but it cannot be used to empirically assess whether biomarker-negative patients might benefit as well. The stratified design randomizes all patients but conducts the randomization separately with each of the biomarker-positive and -negative groups to ensure balance of the treatment arms within each group. This approach provides maximum information about the ability of the biomarker to identify patients who will benefit from the new therapy. A stratified design does not allow the biomarker to influence what treatment a patient receives. This can be an advantage in a situation where there is some uncertainty about the strength of a biomarker?s performance because there were limited specimens available on which to perform preliminary validations during the biomarker development process. The strategy design randomizes patients between no use of the biomarker (all patients receive standard therapy on that arm) and a biomarker-based strategy where biomarker-negative patients are directed to standard therapy and biomarker-positive patients are directed to the new therapy. A strategy design in the context of a single biomarker is particularly inefficient because patients who are negative for the biomarker will receive standard therapy regardless of whether they are randomized to use the biomarker. Due to this inefficiency, this strategy design is generally not recommended in a simple single-biomarker setting. Each of these designs has its advantages and disadvantages; the optimal choice depends on feasibility and what properties have already been established for the biomarker.

Many of the same principles discussed for phase III trials also apply to phase II trials. Some ways in which phase II designs may differ from phase III designs include alternative or ?earlier? endpoints (for example, disease progression or tumor response) and the possibility of non-randomized (for example, single-arm) trials [89]. Just as for drug trials, phase II designs incorporating biomarkers are generally not definitive and serve mostly as a screen to determine whether there is sufficient promise to proceed to a phase III trial. A recently proposed randomized biomarker-based phase II trial design has as its primary aim the generation of sufficient data to inform the decision about the best design for a subsequent phase III trial [90].

Lastly, the same basic design considerations for trials incorporating single biomarkers apply to omics tests, even though it can be more difficult to properly evaluate the body of evidence for an omics test to determine its readiness for use in a clinical trial. The difficulties lie in the complexity of some predictors and the generally incomplete reporting of methods and results for such studies. By considering the criteria presented here, it is hoped that the body of evidence will be more systematically and thoroughly reviewed before omics tests are applied in clinical trials where they will be used to guide treatment decisions.

To prepare for a prospective phase II or phase III trial that will use an omics test, a thorough review should be conducted of all retrospective validation studies of the test to assess the evidence for both its prognostic value and its predictive ability. This review should be undertaken before it is proposed that a prospective clinical trial be conducted to definitively evaluate the clinical benefit of the test (clinical utility). ?Prognostic? refers to the ability of a test to predict clinical outcome in the absence of therapy (that is, natural history) or in the presence of a standard therapy that all patients are likely to receive. ?Predictive? (also called treatment effect modifier, treatment selection, or treatment guiding) refers to the ability of a test to predict benefit or lack of benefit (potentially even harm) from a particular therapy relative to other available therapies. Most developmental studies provide evidence of only some prognostic value of a predictor but do not provide convincing evidence of its predictive value, which is best assessed in the context of a randomized trial. Even a prospective-retrospective study might not be an option to establish predictive utility if there are no specimens available from a trial with the relevant and well-controlled treatment randomization.

In some situations, when the prognostic value of a test has been established as sufficiently robust in retrospective studies, the test can be used in a prospective clinical trial to limit the group of patients who should be randomized. An example of such a situation is provided by the TAILORx (Trial Assigning IndividuaLized Options for Treatment; NCT00310180) trial in patients with node-negative, hormone receptor-positive, HER2-negative breast cancer [91,92]. That adjuvant trial tested more than 10,000 tumors for the 21-gene recurrence score, assigning patients with low-risk scores to adjuvant endocrine treatment and those with high-risk scores to standard-of-care adjuvant chemotherapy or adjuvant chemotherapy treatment trials, in addition to adjuvant endocrine treatment. Patients with intermediate-risk scores were randomized to receive endocrine therapy with or without chemotherapy as adjuvant treatment. It was thought to be firmly established that the risk of recurrence for patients with a value of the 21-gene recurrence score in the low-risk range was so small that those patients had very little potential to benefit from the addition of chemotherapy to hormonal therapy. This conclusion was supported by high-quality evidence from a prospective-retrospective study conducted with banked specimens from the tamoxifen arm of a large clinical trial [93], and additional confirmation was provided in a subsequent case-control study [94]. Information about the benefit of chemotherapy for patients with risk scores in the intermediate range was considered to be inconclusive, and the absolute risk of recurrence for those intermediate-risk patients was still fairly favorable; thus it was believed that there was sufficient equipoise about the benefit of chemotherapy in the intermediate-risk group to randomize those patients.

Another frequently encountered situation is one in which an omics test is developed to identify patients who will benefit from a new therapy. Often there is little information about the potential benefit of the new therapy in patients who test ?negative? for the omics test, because the development studies have honed in quickly on the test-positive cases. To provide the most rigorous assessment of the ability of the omics test to predict benefit of the new treatment, one should ideally also randomize test-negative patients to receive or not receive the new therapy. For randomization of test-negative patients to be considered ethical, however, there must be careful examination for evidence of any potential risks, including not only risks due to toxicities of the new therapy but also any risk of receiving an ineffective new therapy in lieu of an established effective therapy, if that is the randomization being proposed. If a trial will randomize test-negative patients, there should be provisions for aggressive futility monitoring so that the trial can be stopped early if substantial evidence emerges that these patients are not benefitting from the new therapy.

23. Develop a clinical trial protocol that contains clearly stated objectives and methods and an analysis plan that includes justification of sample size; lock down and fully document all aspects of the omics test and establish analytical validation of the predictor.

A clinical trial to evaluate the clinical utility of an omics test should be conducted with just as much rigor as a clinical trial to evaluate a new therapy. This includes development of a formal protocol clearly detailing pre-specified hypotheses, study methods, and a statistical analysis plan. Like the formulation of a new drug, all aspects of the omics test should be clearly specified, either in the main protocol or as a supplement to the protocol. The information to be documented includes details of the specimen requirements, assay SOPs, data quality assessments, data pre-processing, specification of the mathematical predictor model, and interpretation and reporting of the output of the predictor model for clinical decision making. The omics test must be analytically validated in accordance with the relevant regulatory requirements (see ?Ethical, legal, and regulatory issues?) before it is used in a trial where it will influence patient care. International working groups such as the International Conference on Harmonisation of Technical Requirements for Registration of Pharmaceuticals for Human Use [95] have issued several guidance documents outlining the principles of good clinical practice and statistical principles for clinical trials (Guidelines E6-R1, E8, E9, and E10); investigators are also expected to adhere to these principles when conducting clinical trials involving the use of omics tests.

24. Establish a secure clinical database so that links among clinical data, omics data, and predictor results remain appropriately blinded, under the control of the study statistician.

Good clinical practice requires that clinical data be maintained in a secure clinical database with access controls and quality assurance procedures in place to ensure data integrity. The same good practices should be followed for maintaining and managing the omics data and predictor results. Blinding should be maintained under the control of the study statistician and linkages made between the clinical data and omics test results only when needed for the protocol-specified interim monitoring and for the final definitive analysis. Investigators are expected to adhere to the principles outlined in the FDA *Guidance for Industry: Computerized Systems Used in Clinical Investigations*[82].

25. Include in the protocol the names of the primary individuals who are responsible for each aspect of the study.

Successful omics-based clinical research requires an interdisciplinary team of experts, generally including laboratory scientists, clinicians, pathologists, statisticians, bioinformaticians, database developers, computational scientists, data managers, and regulatory experts. A standard therapy trial team may not sufficiently cover all of these areas of expertise. It is important that the need for these varied types of expertise is fully recognized and that involvement of the essential individuals is documented by naming the specific responsible individuals in the protocol document.

### Ethical, legal, and regulatory issues

26. Establish communication with the individuals, offices, and agencies that will oversee the ethical, legal, and regulatory issues that are relevant to the conduct of the trial.

Legal, ethical, and regulatory issues must be considered for an omics test to proceed from the research laboratory to the clinic. In particular, the developer needs to consider federal, state, and local laws regarding human participant protection issues [96]; the environment and procedures necessary for performing *in vitro* diagnostic tests; and intellectual property considerations for the test, specimens omics assay platform, and computer software. Careful attention must be paid to the potential for real or perceived conflicts of interest on the part of investigators, institutions, and sponsors. Appropriate safeguards and oversight must be in place; potential risks and disclosures of conflicts of interest must be clearly acknowledged (see Criterion 27). Navigation of the ethical, legal, and regulatory aspects of using an omics predictor in a clinical trial is best approached with a team [83]. Roles of relevant individuals, offices, and institutions should be clearly defined at the outset. Within the primary institution this will probably include a Principal Investigator, a Trial Sponsor, an Institutional Review Board, and a Technology Transfer Office (TTO). External contacts might include institutes within the National Institutes of Health or other funding agency, a Cooperative Clinical Trials Group, commercial partners, and the therapeutic product and medical device divisions of the FDA (Center for Biologics Evaluation and Research, Center for Drug Evaluation and Research, Center for Devices and Radiological Health). All members of the team have an interest and responsibility to ensure that ethical, legal, and regulatory requirements for using the omics predictor in the context of a clinical trial are comprehensively addressed.

27. Ensure that the informed consent documents to be signed by study participants accurately describe the risks and potential benefits associated with use of the omics test and include provisions for banking of specimens, particularly to allow for ?bridging studies? to validate new or improved assays.

When an omics test is to be used in a clinical trial where it will guide patient care or require that a patient undergo a procedure that is not part of standard care, appropriate informed consent must be obtained. The consent forms must accurately describe the risks and potential benefits associated with use of the test (and state that the test itself is investigational, if it has not received FDA approval or clearance). All potential conflicts of interest on the part of the study investigators or sponsoring institutions must also be disclosed in the informed consent documents. The participant must be informed of the degree of likelihood that the test result will be wrong (and how frequently this is expected to occur), potentially resulting in inaccurate treatment assignment. The participant should understand the likely consequences should an erroneous treatment assignment occur. Potential adverse events from the testing process, for instance, from an invasive tissue biopsy, must be clearly explained. Informed consent should also address banking specimens to allow for ?bridging studies? that use the specimens collected during the trial to subsequently validate new or improved assays. The investigator is responsible for ensuring that the use of an omics predictor in a clinical trial is reviewed by the responsible parties at participating institutions (for example, Institutional Review Board, protocol review committee), trial sponsors (for example, National Cancer Institute, universities, companies), and the FDA (for example, through Investigational Device Exemption (IDE) and/or Investigational New Drug applications; see Criterion 30).

28. Address any intellectual property issues regarding the use of the specimens, biomarkers, assays, and computer software used for calculation of the predictor.

The investigator should address any intellectual property issues for use of the specimens, biomarkers, assays, and computer software used for calculation of the predictor. Intellectual property claims can apply to many aspects of an omics test. For instance, the claims can attach to the specimens used to develop the test, the analyte(s) being measured, the assays themselves or components thereof, and/or the computer software used for calculation of the test result. Before developing the test, it is wise to investigate all potential sources of intellectual property to determine whether any rights exist. It is likewise advisable to anticipate any intellectual property that may be generated in the development process and to agree in advance how it will be designated. All existing property rights and agreements concerning future rights should be clearly documented and distributed to all interested parties as early as is practical. The institutional TTO should determine early in the course of assay development whether there are patents on intended biomarkers or the assays to measure them (or components of the assays) that could restrict freedom to operate or develop the test. If there are such patents, the TTO and investigator should contact the patent holder early and determine whether licensure or other accommodations may be made to enable further development. For example, the National Cancer Institute's Cancer Therapy Evaluation Program (CTEP) has agreements with pharmaceutical companies concerning the use of specimens from CTEP-sponsored trials in which the companies have certain rights to any inventions that derive from specimens from patients who were treated with the company?s agent. The TTO and investigator should inquire about these rights when considering use of specimens from such trials. More information is available at the CTEP website [97].

29. Ensure that the omics test is performed in a Clinical Laboratory Improvement Amendments-certified laboratory if the results will be used to determine treatment or will be reported to the patient or the patient?s physician at any time, even after the trial has ended or the patient is no longer participating in the study.

The assay developer is responsible for ensuring that the omics test is performed in an appropriate environment with adherence to good laboratory practice. If test results will be reported to the patient or the patient?s physician at any time, even after the patient comes off the study, the assay must at least be performed in a CLIA-certified laboratory [98]. CLIA was enacted by Congress in 1988, and thus this requirement is federal law. CLIA is administered by the Centers for Medicare and Medicaid Services. There may also be applicable state laws imposing additional requirements that must be followed for the performance of *in vitro* diagnostic tests [99-103].

30. Ensure that appropriate regulatory approvals have been obtained for investigational use of the omics test. If a prospective trial is planned in which the test will guide treatment, consider a pre-submission consultation with the US Food and Drug Administration.

Federal regulations for investigational and clinical use of *in vitro* diagnostics also apply to omics tests. The investigator should contact the FDA early in the planning stages of a trial that will use an omics test to ascertain whether an IDE must be filed [104]. IDE regulations [102] apply to any device, as defined by the FDA [103], that poses significant risk. An IDE is designed to allow the collection of safety and effectiveness data to support further development toward marketing the device and may be required before an omics predictor can be used in a clinical trial. For predictive tests, an IDE for the predictor may be requested as part of the Investigational New Drug application [100] for the companion drug. A device review considers the omics assay as well as other aspects of use of the test, including procedures required to obtain the specimen, the mathematical predictor model, and the format of the results report. It is advisable to obtain a Pre-Sub (formerly known as a pre-IDE) consultation with the Office of In Vitro Diagnostic Device Evaluation and Safety in the Center for Devices and Radiologic Health of the FDA. A Pre-Sub is a free, non-binding consultation with FDA personnel that can help determine the regulatory mechanism, if any, that best suits the development plan [103,104].

Regulatory classifications are determined by intended use and potential risk. Pertinent risks associated with use of a device could be related to specimen collection procedure (for example, risk of a biopsy), performance of the test, or effects of therapy indicated by results produced by the test, but also could be social or psychological, depending on the intended use of the test. The FDA uses several classifications that may apply to an omics predictor. An In Vitro Diagnostic Multivariate Index Assay refers to any assay that uses multiple variables to yield a patient-specific result whose derivation is not easily verifiable by the end user [101]. The FDA recently introduced a Biomarker Qualification mechanism [105] to streamline the scientific development of biomarkers and their use in the drug development process. The qualification of a biomarker is independent of the specific assay used to measure it, although at least one reliable assay must be available. Once qualified, the biomarker can be used to develop other drugs or assays without the need to reestablish the validity of using that biomarker in the same context of use. However, qualification of a biomarker does not eliminate the need to satisfy other regulatory requirements for use of the biomarker test in patient care, such as an IDE for investigational use or clearance or approval for marketing. Early consultation with the FDA about which mechanism is most appropriate can help streamline the regulatory approval process.

## Summary

Evaluation of the readiness of an omics test to be used in clinical care or in a trial where it will guide patient therapy requires careful consideration of the body of evidence supporting the test?s potential clinical utility and safety, as well as an understanding of ethical, legal, and regulatory issues. Considerations include those relating to specimens, assays, the appropriateness of the statistical methods used to develop and validate the omics test, the principles of clinical study design, and regulatory, ethical, and legal issues. It is hoped that the 30-point checklist presented here will help investigators to more reliably evaluate the quality of evidence in support of omics tests, to understand what information is important to document about data provenance and the test development process, and to plan appropriately for the use of omics predictors in clinical trials or clinical care, and that it will guide them toward the use of best practices in omics test development. The ultimate goal is to develop a more efficient and reliable process to move omics assays from promising research results to clinically useful tests that improve patient care and outcomes.

## Abbreviations

CLIA: Clinical Laboratory Improvement Amendments; CTEP: Cancer Therapy Evaluation Program; FDA: Food and Drug Administration; IDE: Investigational Device Exemption; IOM: Institute of Medicine; RIN: RNA Integrity Number; SOP: standard operating procedure; TTO: Technology Transfer Office.

## Competing interests

The authors declare that they have no competing interests.

## Authors? contributions

BAC and LMM conceived the idea for this paper and the checklist. The initial draft of the manuscript was a joint effort among several authors contributing according to their particular areas of expertise (WLB, MMC, BAC, DAE, TGL, and LMM). All authors provided comments, suggested edits, and contributed additional expertise to enhance the initial draft and produce the final version of the manuscript. All authors read and approved the final manuscript.
